# TOPSIS Method for Probabilistic Linguistic MAGDM with Entropy Weight and Its Application to Supplier Selection of New Agricultural Machinery Products

**DOI:** 10.3390/e21100953

**Published:** 2019-09-29

**Authors:** Jianping Lu, Cun Wei, Jiang Wu, Guiwu Wei

**Affiliations:** 1 School of Business, Sichuan Normal University, Chengdu 610101, China; lujp2002@163.com; 2 School of Statistics, Southwestern University of Finance and Economics, Chengdu 611130, China; wujiang@swufe.edu.cn; 3 Communications Systems and Networks (CSN) Research Group, Department of Electrical and Computer Engineering, Faculty of Engineering, King Abdulaziz University, Jeddah 21589, Saudi Arabia

**Keywords:** multiple attribute group decision making (MAGDM), probabilistic linguistic term sets (PLTSs), information entropy, TOPSIS method, supplier selection, new agricultural machinery products

## Abstract

In multiple attribute group decision making (MAGDM) problems, uncertain decision information is well-represented by linguistic term sets (LTSs). These LTSs are easily converted into probabilistic linguistic sets (PLTSs). In this paper, a TOPSIS method is proposed for probabilistic linguistic MAGDM in which the attribute weights are completely unknown, and the decision information is in the form of probabilistic linguistic numbers (PLNs). First, the definition of the scoring function is used to solve the probabilistic linguistic entropy, which is then employed to objectively derive the attribute weights. Second, the optimal alternatives are determined by calculating the shortest distance from the probabilistic linguistic positive ideal solution (PLPIS) and on the other side the farthest distance of the probabilistic linguistic negative ideal solution (PLNIS). This proposed method extends the applications range of the traditional entropy-weighted method. Moreover, it doesn’t need the decision-maker to give the attribute weights in advance. Finally, a numerical example for supplier selection of new agricultural machinery products is used to illustrate the use of the proposed method. The result shows the approach is simple, effective and easy to calculate. The proposed method can contribute to the selection of suitable alternative successfully in other selection problems.

## 1. Introduction

In many decision-making problems, it has been traditionally supposed that all information is depicted in the form of crisp numbers. However, most of a decision makers’ assessment information is imprecise or uncertain [1,2,3]. Hence, he or she can’t express his or her preferences using an exact numerical value [4,5,6,7]. In order to depict the qualitative assessment information easily, Herrera and Martinez [8] gave the linguistic term sets (LTSs) for computing with words. Herrera and Martinez [9] combined linguistic and numerical information on the basis of the two-tuple fuzzy linguistic representation model. Herrera and Martinez [10] defined the linguistic two-tuples for handling multigranular hierarchical linguistic contexts. Dong and Herrera-Viedma [11] tackled the consistency-driven automatic method to interval numerical scales of LTSs for linguistic GDM with preference relation. Recently, two-tuple linguistic processing model are extended to interval numbers [12,13], intuitionistic fuzzy sets [14,15,16], hesitant fuzzy sets [17,18,19,20], and bipolar fuzzy sets [21,22]. Furthermore, Rodriguez, et al. [23] defined the hesitant fuzzy linguistic term sets (HFLTSs) on the basis of hesitant fuzzy sets [24] and linguistic term sets [25] which allow DMs to provide several possible linguistic variable. However, in most of the current researches on HFLTSs, all possible values supplied by the DMs have equal weight or importance. Obviously, it is inconsistent with the reality. In both personal MADM and multiple attribute group decision making (MAGDM) problems, the DMs may offer possible linguistic terms so that these offered possible values may have different probability distributions. Thus, Pang, et al. [26] proposed the probabilistic linguistic term sets (PLTSs) to overcome this defect and constructed a framework for ranking PLTSs with the score or deviation degree of each PLTS. Bai, et al. [27] gave a comparison method and proposed a more efficient way to tackle PLTSs. Kobina, et al. [28] proposed some probabilistic linguistic power operators for MAGDM with classical power aggregation operators [29,30,31]. Liang, et al. [32] developed the probabilistic linguistic grey relational analysis (PL-GRA) for MAGDM based on geometric Bonferroni mean [33,34,35,36]. Liao, et al. [37] defined the linear programming method to deal with the MADM with probabilistic linguistic information. Lin, et al. [38] proposed the ELECTRE II method to deal with PLTSs for edge computing. Liao, et al. [39] studied the novel operations of PLTSs to solve the probabilistic linguistic ELECTRE III method. Feng, et al. [40] proposed the probabilistic linguistic QUALIFLEX method with possibility degree comparison. Chen, et al. [41] employed the probabilistic linguistic MULTIMOORA for cloud-based ERP system selection.

Entropy is a very important and efficient tool for measuring uncertain information. The fuzzy entropy was first defined by Zadeh [42]. The beginning point for the cross entropy method is information theory as proposed by Shannon [43]. Kullback and Leibler [44] developed “cross entropy distance” measure between two probability distributions. Furtan [45] studied the entropy theory in firm decision-making. Dhar, et al. [46] investigated the investment decision making with entropy reduction. Yang and Qiu [47] researched the decision-making method based on expected utility and entropy. Muley and Bajaj [48] used the entropy-based approach to solve the fuzzy MADM. Xu and Hu [49] proposed the entropy-based procedures for intuitionistic fuzzy MADM. Chen, et al. [50] constructed concretely an interval-valued intuitionistic fuzzy entropy. Lotfi and Fallahnejad [51] proposed the imprecise Shannon’s entropy in MADM. Khaleie and Fasanghari [52] investigated the intuitionistic fuzzy MAGDM method by using entropy and association coefficient. Zhao, et al. [53] extended the VIKOR method based on cross-entropy for interval-valued intuitionistic fuzzy MAGDM. Peng, et al. [54] defined the cross-entropy for intuitionistic hesitant fuzzy sets in MADM. Tao, et al. [55] developed the entropy measures for linguistic information in MAGDM. Song, et al. [56] studied the MADM for dual uncertain information on the basis of grey incidence analysis and grey relative entropy optimization. Farhadinia and Xu [57] tackled the hesitant fuzzy linguistic entropy and cross-entropy measures in MADM. Xue, et al. [58] proposed the Pythagorean fuzzy LINMAP method with the entropy theory for railway project investment. Biswas and Sarkar [59] developed the Pythagorean fuzzy TOPSIS for MAGDM with unknown weight information through entropy measure. Xiao [60] gave the MADM model based on D-numbers and belief entropy. Xu and Luo [61] defined the information entropy risk measure for large group decision-making method.

TOPSIS (Technique for order performance by similarity to ideal solution) method was initially proposed by Hwang and Yoon [62] for solving a MADM problem, which concentrates on choosing the alternative with the smallest distance from the positive ideal solution (PIS) and with the longest distance from the negative ideal solution (NIS). In the recent years, many scholars indulged in MADM or MAGDM problem based on the TOPSIS method [63,64,65,66,67,68]. The goal of this paper is to extend the TOPSIS method to solve the probabilistic linguistic MAGDM with unknown weight information on the basis of the information entropy. The innovativeness of the paper can be summarized as follows: (1) the TOPSIS method is extended by PLTSs with unknown weight information; (2) the probabilistic linguistic TOPSIS (PL-TOPSIS) method is proposed to solve the probabilistic linguistic MAGDM problems with entropy weight; (3) a case study for supplier selection of new agricultural machinery products is supplied to show the developed approach; and (4) some comparative studies are provided with the probabilistic linguistic weighted average (PLWA) operator and the PL-GRA method to give effect to the rationality of PL-TOPSIS method.

The remainder of this paper is set out as follows. Section 2 supplies some basic concepts of PLTSs. In Section 3, the TOPSIS method is proposed for probabilistic linguistic MAGDM problems with entropy weight. In Section 4, a case study for supplier selection of new agricultural machinery products is given and some comparative analysis is conducted. The study finishes with some conclusions in Section 5.

## 2. Preliminaries

In this section, we review some concepts and operations related to linguistic terms sets and PLTSs.

**Definition** **1.** ([26]) *Let $L=\left\{l_{\alpha }|\alpha =-\theta ,\cdots ,-2,-1,0,1,2,\cdots \theta \right\}$ be an LTS, the linguistic terms $l_{\alpha }$ can express the equivalent information to β which is expressed with the transformation function g:*

(1) $$g:\left[l_{-\theta },l_{\theta }\right]\to \left[0,1\right],\;g\left(l_{\alpha }\right)=\frac{\alpha +\theta }{2\theta }=\beta$$

*β can also be expressed the equivalent information to the linguistic terms $l_{\alpha }$ which is denoted with the transformation function $g^{-1}$:*

(2) $$g^{-1}:\left[0,1\right]\to \left[l_{-\theta },l_{\theta }\right],\;g^{-1}\left(\beta \right)=l_{\left(2\beta -1\right)\theta }=l_{\alpha }$$

Pang, Wang and Xu [26] proposed a novel concept called probabilistic linguistic term sets to depict qualitative information.

**Definition** **2.** ([26]) *Given an LTS $L=\left\{l_{\alpha }|\alpha =-\theta ,\cdots ,-2,-1,0,1,2,\cdots \theta \right\}$, a PLTS is defined as:*

(3) $$L\left(p\right)=\left\{l^{\left(\phi \right)}\left(p^{\left(\phi \right)}\right)|l^{\left(\phi \right)}\in L,p^{\left(\phi \right)}\geq 0,\;\phi =1,2,\cdots ,\#L\left(p\right),\sum_{\phi =1}^{\#L\left(p\right)}p^{\left(\phi \right)}\leq 1\right\}$$

*where $l^{\left(\phi \right)}\left(p^{\left(\phi \right)}\right)$ is the ϕth linguistic term $l^{\left(\phi \right)}$ associated with the probability value $p^{\left(\phi \right)}$, and #L(p) is the length of linguistic terms in L(p). The linguistic term $l^{\left(\phi \right)}$ in L(p) are arranged in ascending order.*

In order to easy computation, Pang, Wang and Xu [26] normalized the PLTS L(p) as $\tilde{L}\left(p\right)=\left\{l^{\left(\phi \right)}\left({\tilde{p}}^{\left(\phi \right)}\right)|l^{\left(\phi \right)}\in L,{\tilde{p}}^{\left(\phi \right)}\geq 0,\;\phi =1,2,\cdots ,\#L\left(\tilde{p}\right),\sum_{\phi =1}^{\#L\left(p\right)}{\tilde{p}}^{\left(\phi \right)}=1\right\}$, where ${\tilde{p}}^{\left(\phi \right)}=p^{\left(\phi \right)}/\sum_{\phi =1}^{\#L\left(p\right)}p^{\left(\phi \right)}$ for all $\phi =1,2,\cdots ,\#L\left(\tilde{p}\right)$.

Moreover, in the process of MAGDM issue, the numbers of linguistic terms in PLTSs are always different, which brings great trouble to calculate. In this case, we need to increase the numbers of linguistic terms for the PLTSs in which the numbers of linguistic terms are relatively small, so that they have the same number of linguistic terms.

**Definition** **3.** ([26]) *Let $L=\left\{l_{\alpha }|\alpha =-\theta ,\cdots ,-1,0,1,\cdots \theta \right\}$ be an LTS, ${\tilde{L}}_{1}\left(\tilde{p}\right)=\left\{{l_{1}}^{\left(\phi \right)}\left({{\tilde{p}}_{1}}^{\left(\phi \right)}\right)|\;\phi =1,2,\cdots ,\#{\tilde{L}}_{1}\left(\tilde{p}\right)\right\}$ and ${\tilde{L}}_{2}\left(\tilde{p}\right)=\left\{{l_{2}}^{\left(\phi \right)}\left({{\tilde{p}}_{2}}^{\left(\phi \right)}\right)|\;\phi =1,2,\cdots ,\#{\tilde{L}}_{2}\left(\tilde{p}\right)\right\}$ be two PLTSs, where $\#{\tilde{L}}_{1}\left(\tilde{p}\right)$ and $\#{\tilde{L}}_{2}\left(\tilde{p}\right)$ are the numbers of PLTS ${\tilde{L}}_{1}\left(\tilde{p}\right)$ and ${\tilde{L}}_{2}\left(\tilde{p}\right)$, respectively. If $\#{\tilde{L}}_{1}\left(\tilde{p}\right)>\#{\tilde{L}}_{2}\left(\tilde{p}\right)$, then add $\#{\tilde{L}}_{1}\left(\tilde{p}\right)-\#{\tilde{L}}_{2}\left(\tilde{p}\right)$ linguistic terms to ${\tilde{L}}_{2}\left(\tilde{p}\right)$. Moreover, the added linguistic terms should be the smallest linguistic term in ${\tilde{L}}_{2}\left(\tilde{p}\right)$ and the probabilities of added linguistic terms should be zero.*

After we have defined the concept of PLTSs, we need to propose a method to compare these PLTSs. In order to do so, in the following, we first define the score and deviation degree of PLTSs.

**Definition** **4.** ([26]) *For a PLTS $\tilde{L}\left(\tilde{p}\right)=\left\{l^{\left(\phi \right)}\left({\tilde{p}}^{\left(\phi \right)}\right)|\;\phi =1,2,\cdots ,\#\tilde{L}\left(\tilde{p}\right)\right\}$, the expected value $E\left(\tilde{L}\left(\tilde{p}\right)\right)$ and deviation degree $\sigma \left(\tilde{L}\left(\tilde{p}\right)\right)$ of $\tilde{L}\left(\tilde{p}\right)$ is defined:*

(4) $$E\left(\tilde{L}\left(\tilde{p}\right)\right)=\sum_{\phi =1}^{\#\tilde{L}\left(\tilde{p}\right)}g\left(l^{\left(\phi \right)}\right){\tilde{p}}^{\left(\phi \right)}/\sum_{\phi =1}^{\#\tilde{L}\left(\tilde{p}\right)}{\tilde{p}}^{\left(\phi \right)}$$

(5) $$\sigma \left(\tilde{L}\left(\tilde{p}\right)\right)=\sqrt{\sum_{\phi =1}^{\#\tilde{L}\left(\tilde{p}\right)}{\left(g\left(l^{\left(\phi \right)}\right){\tilde{p}}^{\left(\phi \right)}-E\left(\tilde{L}\left(\tilde{p}\right)\right)\right)}^{2}}/\sum_{\phi =1}^{\#\tilde{L}\left(\tilde{p}\right)}{\tilde{p}}^{\left(\phi \right)}$$

By using the Equations (4) and (5), the order relation between two PLTSs is defined as: (1) if $E\left({\tilde{L}}_{1}\left(\tilde{p}\right)\right)>E\left({\tilde{L}}_{2}\left(\tilde{p}\right)\right)$, then ${\tilde{L}}_{1}\left(\tilde{p}\right)>{\tilde{L}}_{2}\left(\tilde{p}\right)$; and (2) if $E\left({\tilde{L}}_{1}\left(\tilde{p}\right)\right)=E\left({\tilde{L}}_{2}\left(\tilde{p}\right)\right)$, then if $\sigma \left({\tilde{L}}_{1}\left(\tilde{p}\right)\right)=\sigma \left({\tilde{L}}_{2}\left(\tilde{p}\right)\right)$, then ${\tilde{L}}_{1}\left(\tilde{p}\right)={\tilde{L}}_{2}\left(\tilde{p}\right)$; if $\sigma \left({\tilde{L}}_{1}\left(\tilde{p}\right)\right)<\sigma \left({\tilde{L}}_{2}\left(\tilde{p}\right)\right)$, then, ${\tilde{L}}_{1}\left(\tilde{p}\right)>{\tilde{L}}_{2}\left(\tilde{p}\right)$.

**Definition** **5.** ([69]) *Let $L=\left\{l_{\alpha }|\alpha =-\theta ,\cdots ,-1,0,1,\cdots \theta \right\}$ be an LTS. And let ${\tilde{L}}_{1}\left(\tilde{p}\right)=\left\{{l_{1}}^{\left(\phi \right)}\left({{\tilde{p}}_{1}}^{\left(\phi \right)}\right)|\;\phi =1,2,\cdots ,\#{\tilde{L}}_{1}\left(\tilde{p}\right)\right\}$ and ${\tilde{L}}_{2}\left(\tilde{p}\right)=\left\{{l_{2}}^{\left(\phi \right)}\left({{\tilde{p}}_{2}}^{\left(\phi \right)}\right)|\;\phi =1,2,\cdots ,\#{\tilde{L}}_{2}\left(\tilde{p}\right)\right\}$ be two PLTSs with $\#{\tilde{L}}_{1}\left(\tilde{p}\right)=\#{\tilde{L}}_{2}\left(\tilde{p}\right)$, then Hamming distance $d\left({\tilde{L}}_{1}\left(\tilde{p}\right),{\tilde{L}}_{2}\left(\tilde{p}\right)\right)$ between ${\tilde{L}}_{1}\left(\tilde{p}\right)$ and ${\tilde{L}}_{2}\left(\tilde{p}\right)$ is defined as follows:*

(6) $$d\left({\tilde{L}}_{1}\left(\tilde{p}\right),{\tilde{L}}_{2}\left(\tilde{p}\right)\right)=\frac{\sum_{\phi =1}^{\#{\tilde{L}}_{1}\left(\tilde{p}\right)}\left|{{\tilde{p}}_{1}}^{\left(\phi \right)}g\left({l_{1}}^{\left(\phi \right)}\right)-{{\tilde{p}}_{2}}^{\left(\phi \right)}g\left({l_{2}}^{\left(\phi \right)}\right)\right|}{\#{\tilde{L}}_{1}\left(\tilde{p}\right)}$$

## 3. TOPSIS Method for Probabilistic Linguistic MAGDM with Entropy Weight

In this section, we propose a novel probabilistic linguistic TOPSIS method for MAGDM problems with unknown weight information. The following notations are used to solve the probabilistic linguistic MAGDM problems. Let $A=\left\{A_{1},A_{2},\cdots ,A_{m}\right\}$ be a discrete set of alternatives, and $G=\left\{G_{1},G_{2},\cdots ,G_{n}\right\}$ with weight vector $w=\left(w_{1},w_{2},\cdots ,w_{n}\right)$, where $\omega_{j}\in \left[0,1\right]$, j=1,2,⋯,n, $\sum_{j=1}^{n}w_{j}=1$, and a set of experts $E=\left\{E_{1},E_{2},\cdots ,E_{q}\right\}$. Suppose that there are n qualitative attribute $A=\left\{A_{1},A_{2},\cdots ,A_{m}\right\}$ and their values are evaluated by qualified experts and denoted as linguistic expressions information $l_{ij}^{k}\left(i=1,2,\cdots ,m,j=1,2,\cdots ,n,k=1,2,\cdots ,q\right)$.

Then, the PL-TOPSIS method is designed to solve the MAGDM problems with entropy weight. The detailed calculating steps are given as follows:

**Step 1.** Convert the linguistic information $l_{ij}^{k}\left(i=1,2,\cdots ,m,j=1,2,\cdots ,n,k=1,2,\cdots ,q\right)$ into probabilistic linguistic information ${l_{ij}}^{\left(\phi \right)}\left({p_{ij}}^{\left(\phi \right)}\right),\phi =1,2,\cdots ,\#L_{ij}\left(p\right)$ and construct the probabilistic linguistic decision matrix $L={\left(L_{ij}\left(p\right)\right)}_{m\times n}$, $L_{ij}\left(p\right)=\left\{{l_{ij}}^{\left(\phi \right)}\left({p_{ij}}^{\left(\phi \right)}\right)|\;\phi =1,2,\cdots ,\#L_{ij}\left(p\right)\right\}$ (i=1,2,⋯,m,j=1,2,⋯,n).

**Step 2.** Derive the normalized probabilistic linguistic matrix $\tilde{L}={\left({\tilde{L}}_{ij}\left(\tilde{p}\right)\right)}_{m\times n}$, $L_{ij}\left(\tilde{p}\right)=\left\{{l_{ij}}^{\left(\phi \right)}\left({{\tilde{p}}_{ij}}^{\left(\phi \right)}\right)|\;\phi =1,2,\cdots ,\#L_{ij}\left(\tilde{p}\right)\right\}$ (i=1,2,⋯,m,j=1,2,⋯,n). Thus, probabilistic linguistic information for the alternative $A_{i}\in A$ with respect to the all the attribute G can be expressed as: $PLA_{i}=\left(l_{i1}^{\left(\phi \right)}\left({\tilde{p}}_{i1}^{\left(\phi \right)}\right),l_{i2}^{\left(\phi \right)}\left({\tilde{p}}_{i2}^{\left(\phi \right)}\right),\cdots ,l_{in}^{\left(\phi \right)}\left({\tilde{p}}_{in}^{\left(\phi \right)}\right)\right)$, $\phi =1,2,\cdots ,\#L_{ij}\left(\tilde{p}\right)$.

**Step 3.** Compute the weight values with entropy.

The weight of attributes is very important in decision making problem. Many scholars focus on decision making problems with incomplete or unknown attributes weight information in different fuzzy environment [70,71,72,73,74,75]. Entropy [43] is a conventional term from information theory which is also used to determine weight of attributes. The larger the value of entropy in a given attribute is, the smaller the differences in the ratings of alternatives with respect to this attribute. In turn, this means that this kind of attribute supplies less information and has a smaller weight. Firstly, the normalized decision matrix $NL_{ij}\left(\tilde{p}\right)$ is derived as follows:

(7) $$NL_{ij}\left(\tilde{p}\right)=\frac{\sum_{\phi =1}^{\#{\tilde{L}}_{1}\left(\tilde{p}\right)}\left({{\tilde{p}}_{ij}}^{\left(\phi \right)}g\left({l_{ij}}^{\left(\phi \right)}\right)\right)}{\sum_{i=1}^{m}\sum_{\phi =1}^{\#{\tilde{L}}_{1}\left(\tilde{p}\right)}\left({{\tilde{p}}_{ij}}^{\left(\phi \right)}g\left({l_{ij}}^{\left(\phi \right)}\right)\right)},\;j=1,2,\cdots ,n$$

Then, the information of Shannon entropy $E=\left(E_{1},E_{2},\cdots ,E_{n}\right)$ is calculated as follows:

(8) $$E_{j}=-\frac{1}{\ln m}\sum_{i=1}^{m}NL_{ij}\left(\tilde{p}\right)\ln NL_{ij}\left(\tilde{p}\right)$$

and $NL_{ij}\left(\tilde{p}\right)\ln NL_{ij}\left(\tilde{p}\right)$ is defined as 0, if $NL_{ij}\left(\tilde{p}\right)=0$.

Finally, the vector of attribute weights $w=\left(w_{1},w_{2},\cdots ,w_{n}\right)$ is computed:

(9) $$w_{j}=\frac{1-E_{j}}{\sum_{j=1}^{n}\left(1-E_{j}\right)},\;j=1,2,\cdots ,n.$$

**Step 4**. Define the probabilistic linguistic positive ideal solution (PLPIS) and probabilistic linguistic negative ideal solution (PLNIS):

(10) $$PLPIS=\left(PLPIS_{1},PLPIS_{2},\cdots ,PLPIS_{n}\right)$$

(11) $$PLNIS=\left(PLNIS_{1},PLNIS_{2},\cdots ,PLNIS_{n}\right)$$

where

(12) $$PLPIS_{j}=\left\{pl_{j}^{\left(\phi \right)}\left({\tilde{p}}_{j}^{\left(\phi \right)}\right)|\;\phi =1,2,\cdots ,\#L_{ij}\left(\tilde{p}\right)\right\},\;E\left(PLPIS_{j}\right)=\left\{\underset{i}{\max}E\left(L_{ij}\left(\tilde{p}\right)\right)\right\}$$

(13) $$PLNIS_{j}=\left\{nl_{j}^{\left(\phi \right)}\left({\tilde{p}}_{j}^{\left(\phi \right)}\right)|\;\phi =1,2,\cdots ,\#L_{ij}\left(\tilde{p}\right)\right\},E\left(PLNIS_{j}\right)=\left\{\underset{i}{\min}E\left(L_{ij}\left(\tilde{p}\right)\right)\right\}$$

**Step 5**. Calculate the distances of each alternative from PLPIS and PLNIS, respectively:

(14) $$d\left(PLA_{i},PLPIS\right)=\sum_{j=1}^{n}w_{j}d\left(PLA_{ij},PLPIS_{j}\right)$$

(15) $$d\left(PLA_{i},PLNIS\right)=\sum_{j=1}^{n}w_{j}d\left(PLA_{ij},PLNIS_{j}\right)$$

(16) $$d\left(PLA_{ij},PLPIS_{j}\right)=\left(\sum_{\phi =1}^{\#L_{ij}\left(\tilde{p}\right)}\left|{p_{ij}}^{\left(\phi \right)}g\left({l_{ij}}^{\left(\phi \right)}\right)-{\tilde{p}}_{j}^{\left(\phi \right)}g\left(pl_{j}^{\left(\phi \right)}\right)\right|\right)/\#L_{ij}\left(\tilde{p}\right)$$

(17) $$d\left(PLA_{ij},PLNIS_{j}\right)=\left(\sum_{\phi =1}^{\#L_{ij}\left(\tilde{p}\right)}\left|{p_{ij}}^{\left(\phi \right)}g\left({l_{ij}}^{\left(\phi \right)}\right)-{\tilde{p}}_{j}^{\left(\phi \right)}g\left(nl_{j}^{\left(\phi \right)}\right)\right|\right)/\#L_{ij}\left(\tilde{p}\right)$$

**Step 6**. Calculate the probabilistic linguistic relative closeness degree (PLRCD) of each alternative from PLPIS.

(18) $$PLRCD\left(PLA_{i},PLPIS\right)=\frac{d\left(PLA_{i},PLNIS\right)}{d\left(PLA_{i},PLPIS\right)+d\left(PLA_{i},PLNIS\right)},\;i=1,2,\cdots ,m$$

**Step 7**. According to the $PLRCD\left(PLA_{i},PLPIS\right)$, the ranking order of all alternatives can be determined. The best alternative is the one closest to PLPIS and farthest from the PLNIS. Thus, if any alternative has the smallest $PLRCD\left(PLA_{i},PLPIS\right)$ value, then, it is the most desirable alternative.

## 4. A Case Study and Comparative Analysis

### 4.1. A Case Study

With the development and improvement of agricultural mechanization and the amendment of preferential farmer policies, the agricultural machinery industry is confronted with broad market prospects for development. The innovation capability has become core competence for an agricultural company. In the process of supply chain integration, agricultural product innovation is being drawn into many corporations, manufacturer, and suppliers instead of manufacturer. From the perspective of manufacturer, the participation of suppliers is significative for the success of new product project. Of most importance, the supplier selection problem in new agricultural machinery products is becoming one of the key issues based on the basic idea of supply chain management. The supplier selection in new agricultural machinery products is a classical MAGDM issue [76,77,78,79,80,81,82]. Thus, in this section we present a numerical example for supplier selection in new agricultural machinery products to illustrate the method proposed in this paper. There is a panel with five possible suppliers of new agricultural machinery products $A_{i}\left(i=1,2,3,4,5\right)$ to select. The experts select four beneficial attribute to evaluate the five possible suppliers of new agricultural machinery products: (1) G_1_ is the supplier’s business reputation; (2) G_2_ is the supplier’s technical capability; (3) G_3_ is the supplier’s experience; and (4) G_4_ is the supplier’s willingness to cooperate. The five possible suppliers of new agricultural machinery products $A_{i}\left(i=1,2,3,4,5\right)$ are to be evaluated by using the linguistic term set $\begin{array}{l} L=\{l_{-3}=extremely\;poor(EP),l_{-2}=very\;poor(VP),\;l_{-1}=poor(P),l_{0}=medium(M),\;\\ l_{1}=good(G),\;l_{2}=very\;good(VG),l_{3}=extremely\;good(EG)\} \end{array}$ by the five decision makers under the above four attributes, as listed in the Table 1, Table 2, Table 3, Table 4 and Table 5.

In the following, we utilize the PL-TOPSIS method developed for supplier selection of new agricultural machinery products.

**Step 1.** Transform the linguistic variables into probabilistic linguistic decision matrix (Table 6).

**Step 2.** Calculate the normalized probabilistic linguistic decision matrix (Table 7).

**Step 3.** Compute the weight values for attributes from Equations (7)–(9): $w_{1}=0.3020,w_{2}=0.1536,w_{3}=0.2856,w_{4}=0.2587$.

**Step 4**. Determine the PLPIS and PLNIS by Equations (10)–(13) (Table 8):

**Step 5**. Calculate the distances $d\left(PLA_{i},PLPIS\right)$ and $d\left(PLA_{i},PLNIS\right)$ of each alternative by Equations (14)–(17), respectively (Table 9):

**Step 6.** Calculating the PLRCD of each alternative from PLPIS by Equation (18) (Table 10).

**Step 7.** According to the $PLRCD\left(PLA_{i},PLPIS\right)\left(i=1,2,3,4,5\right)$, we can rank all the suppliers of new agricultural machinery products. Obviously, the rank is $A_{3}>A_{1}>A_{4}>A_{5}>A_{2}$ and the best supplier among five alternatives is $A_{3}$.

### 4.2. Comparative Analysis

Firstly, we compare our proposed method with probabilistic linguistic weighted average operator [26], then we can get the calculat3e results: $E\left(Z_{1}\left(w\right)\right)=s_{0.0036},\;E\left(Z_{2}\left(w\right)\right)=s_{-0.6638},$ $E\left(Z_{3}\left(w\right)\right)=s_{0.5812},E\left(Z_{4}\left(w\right)\right)=s_{-0.1787},$ and $E\left(Z_{5}\left(w\right)\right)=s_{-0.1273}.$ Furthermore, we can derive the ranking order: $A_{3}>A_{1}>A_{5}>A_{4}>A_{2}.$ Thus, we have the same optimal supplier $A_{3}$.

Then, we compare our proposed method with probabilistic linguistic grey relational analysis method [32] (let ρ=0.5), then we can get the calculating results: $\varepsilon_{1}^{+}=0.6279,\varepsilon_{2}^{+}=0.4457,\varepsilon_{3}^{+}=0.8410,\varepsilon_{4}^{+}=0.5039,\varepsilon_{5}^{+}=0.5364.$ Furthermore, we can derive the ranking order: $A_{3}>A_{1}>A_{5}>A_{4}>A_{2}.$ Thus, we also have the same optimal supplier $A_{3}$.

From the above analysis, it can be seen that these four methods have the same optimal supplier $A_{2}$ and three methods’ ranking results are slightly different. This verifies the method we proposed is reasonable and effective in this paper. These three methods have their advantages: (1) PL-GRA method only emphasis the shape similarity degree from the positive ideal solution; (2) PLWA operator emphasis group influences degree; (3) our proposed PL-TOPSIS method emphasizes the distance closeness degree from the positive and negative ideal solution with entropy weight information; and (4) Pang, Wang and Xu [26] also developed probabilistic linguistic TOPSIS method for MAGDM with maximizing deviation method. However, the employed distance measures for TOPSIS method in this paper [26] are more or less irrational.

## 5. Conclusions

In this paper, we extend the TOPSIS method to the probabilistic linguistic MAGDM with unknown weight information. Firstly, the basic concept, comparative formula, and Hamming distance of PLNs are briefly reviewed. Then, the definition of the scoring function is utilized to tackle the probabilistic linguistic entropy, which is then employed to objectively compute the attribute weights information. Then, the optimal alternative(s) is determined by calculating the shortest distance from the PLPIS and on the other side the farthest distance of the PLNIS. Finally, a practical case study for supplier selection of new agricultural machinery products is supplied to show the proposed approach and some comparative analysis is also designed to verify the applicability in practical MAGDM problems. The main advantages of this paper can be given as follows: (1) the TOPSIS method is extended by PLTSs with unknown weight information; (2) the probabilistic linguistic TOPSIS method is proposed to solve the probabilistic linguistic MAGDM problems with entropy weight; (3) a case study for supplier selection of new agricultural machinery products is supplied to show the developed approach; (4) our proposed PL-TOPSIS method emphasis the distance similarity degree from the positive and negative ideal solution with entropy weight; and (5) In this research, the TOPSIS method, which may be also a resultful MADM or MAGDM method, has been designed to tackle uncertain decision-making problems. In the future, the application of the proposed models and methods with PLTSs needs to be investigated into other uncertain decision making [83,84,85,86,87,88,89,90] and other uncertain and fuzzy environment [91,92,93,94,95,96,97,98].

## Figures and Tables

**Table 1:** Linguistic decision matrix by the first DM.

Alternatives	G_1_	G_2_	G_3_	G_4_
A_1_	M	G	VG	EP
A_2_	VP	EP	P	VP
A_3_	EG	P	G	EG
A_4_	EP	VG	VP	G
A_5_	G	EG	EP	P

**Table 2:** Linguistic decision matrix by the second DM.

Alternatives	G_1_	G_2_	G_3_	G_4_
A_1_	P	VG	VG	VP
A_2_	EP	P	VP	P
A_3_	EG	P	VG	G
A_4_	VP	G	P	EG
A_5_	EG	VG	EP	EP

**Table 3:** Linguistic decision matrix by the third DM.

Alternatives	G_1_	G_2_	G_3_	G_4_
A_1_	EP	EG	VG	P
A_2_	P	VP	EP	VP
A_3_	EG	P	G	EG
A_4_	EP	VG	VP	G
A_5_	VG	G	EP	VP

**Table 4:** Linguistic decision matrix by the fourth DM.

Alternatives	G_1_	G_2_	G_3_	G_4_
A_1_	EP	G	G	P
A_2_	VP	EP	P	P
A_3_	EG	VP	VG	EG
A_4_	VP	G	EP	VG
A_5_	EG	VG	P	P

**Table 5:** Linguistic decision matrix by the fifth DM.

Alternatives	G_1_	G_2_	G_3_	G_4_
A_1_	M	VG	EG	P
A_2_	P	P	EP	EP
A_3_	EG	EP	EG	G
A_4_	EP	VG	P	EG
A_5_	G	EG	VP	EP

**Table 6:** Probabilistic linguistic decision matrix.

**Alternatives**	**G_1_**	**G_2_**
A_1_	$\left\{l_{0}\left(0.4\right),l_{-1}\left(0.2\right),l_{-3}\left(0.4\right)\right\}$	$\left\{l_{1}\left(0.4\right),l_{2}\left(0.4\right),l_{3}\left(0.2\right)\right\}$
A_2_	$\left\{l_{-2}\left(0.4\right),l_{-1}\left(0.4\right),l_{-3}\left(0.2\right)\right\}$	$\left\{l_{-3}\left(0.4\right),l_{-2}\left(0.2\right),l_{-1}\left(0.4\right)\right\}$
A_3_	$\left\{l_{3}\left(1\right)\right\}$	$\left\{l_{-3}\left(0.2\right),l_{-2}\left(0.2\right),l_{-1}\left(0.6\right)\right\}$
A_4_	$\left\{l_{-3}\left(0.6\right),l_{-2}\left(0.4\right)\right\}$	$\left\{l_{1}\left(0.4\right),l_{2}\left(0.6\right)\right\}$
A_5_	$\left\{l_{1}\left(0.4\right),l_{2}\left(0.2\right),l_{3}\left(0.4\right)\right\}$	$\left\{l_{1}\left(0.2\right),l_{2}\left(0.4\right),l_{3}\left(0.4\right)\right\}$
**Alternatives**	**G_3_**	**G_4_**
A_1_	$\left\{l_{1}\left(0.2\right),l_{2}\left(0.6\right),l_{3}\left(0.2\right)\right\}$	$\left\{l_{-3}\left(0.2\right),l_{-2}\left(0.2\right),l_{-1}\left(0.6\right)\right\}$
A_2_	$\left\{l_{-3}\left(0.4\right),l_{-2}\left(0.2\right),l_{-1}\left(0.4\right)\right\}$	$\left\{l_{-3}\left(0.6\right),l_{-1}\left(0.4\right)\right\}$
A_3_	$\left\{l_{1}\left(0.4\right),l_{2}\left(0.4\right),l_{3}\left(0.2\right)\right\}$	$\left\{l_{1}\left(0.4\right),l_{3}\left(0.6\right)\right\}$
A_4_	$\left\{l_{-3}\left(0.2\right),l_{-2}\left(0.4\right),l_{-1}\left(0.4\right)\right\}$	$\left\{l_{1}\left(0.4\right),l_{2}\left(0.2\right),l_{3}\left(0.4\right)\right\}$
A_5_	$\left\{l_{-3}\left(0.6\right),l_{-2}\left(0.2\right),l_{-1}\left(0.2\right)\right\}$	$\left\{l_{-3}\left(0.4\right),l_{-2}\left(0.2\right),l_{-1}\left(0.4\right)\right\}$

**Table 7:** Normalized probabilistic linguistic decision matrix.

**Alternatives**	**G_1_**	**G_2_**
A_1_	$\left\{l_{-3}\left(0.4\right),l_{-1}\left(0.2\right),l_{0}\left(0.4\right)\right\}$	$\left\{l_{1}\left(0.4\right),l_{2}\left(0.4\right),l_{3}\left(0.2\right)\right\}$
A_2_	$\left\{l_{-3}\left(0.2\right),l_{-2}\left(0.4\right),l_{-1}\left(0.4\right)\right\}$	$\left\{l_{-3}\left(0.4\right),l_{-2}\left(0.2\right),l_{-1}\left(0.4\right)\right\}$
A_3_	$\left\{l_{3}\left(0\right),l_{3}\left(0\right),l_{3}\left(1\right)\right\}$	$\left\{l_{-3}\left(0.2\right),l_{-2}\left(0.2\right),l_{-1}\left(0.6\right)\right\}$
A_4_	$\left\{l_{-3}\left(0\right),l_{-3}\left(0.6\right),l_{-2}\left(0.4\right)\right\}$	$\left\{l_{1}\left(0\right),l_{1}\left(0.4\right),l_{2}\left(0.6\right)\right\}$
A_5_	$\left\{l_{1}\left(0.4\right),l_{2}\left(0.2\right),l_{3}\left(0.4\right)\right\}$	$\left\{l_{1}\left(0.2\right),l_{2}\left(0.4\right),l_{3}\left(0.4\right)\right\}$
**Alternatives**	**G_3_**	**G_4_**
A_1_	$\left\{l_{1}\left(0.2\right),l_{2}\left(0.6\right),l_{3}\left(0.2\right)\right\}$	$\left\{l_{-3}\left(0.2\right),l_{-2}\left(0.2\right),l_{-1}\left(0.6\right)\right\}$
A_2_	$\left\{l_{-3}\left(0.4\right),l_{-2}\left(0.2\right),l_{-1}\left(0.4\right)\right\}$	$\left\{l_{-3}\left(0\right),l_{-3}\left(0.6\right),l_{-1}\left(0.4\right)\right\}$
A_3_	$\left\{l_{1}\left(0.4\right),l_{2}\left(0.4\right),l_{3}\left(0.2\right)\right\}$	$\left\{l_{1}\left(0\right),l_{1}\left(0.4\right),l_{3}\left(0.6\right)\right\}$
A_4_	$\left\{l_{-3}\left(0.2\right),l_{-2}\left(0.4\right),l_{-1}\left(0.4\right)\right\}$	$\left\{l_{1}\left(0.4\right),l_{2}\left(0.2\right),l_{3}\left(0.4\right)\right\}$
A_5_	$\left\{l_{-3}\left(0.6\right),l_{-2}\left(0.2\right),l_{-1}\left(0.2\right)\right\}$	$\left\{l_{-3}\left(0.4\right),l_{-2}\left(0.2\right),l_{-1}\left(0.4\right)\right\}$

**Table 8:** Probabilistic linguistic positive ideal solution (PLPIS) and probabilistic linguistic negative ideal solution (PLNIS).

	G_1_	G_2_
**PLPIS**	$\left\{l_{3}\left(0\right),l_{3}\left(0\right),l_{3}\left(1\right)\right\}$	$\left\{l_{1}\left(0.2\right),l_{2}\left(0.4\right),l_{3}\left(0.4\right)\right\}$
**PLNIS**	$\left\{l_{-3}\left(0\right),l_{-3}\left(0.6\right),l_{-2}\left(0.4\right)\right\}$	$\left\{l_{-3}\left(0.4\right),l_{-2}\left(0.2\right),l_{-1}\left(0.4\right)\right\}$
	**G_3_**	**G_4_**
**PLPIS**	$\left\{l_{1}\left(0.2\right),l_{2}\left(0.6\right),l_{3}\left(0.2\right)\right\}$	$\left\{l_{1}\left(0\right),l_{1}\left(0.4\right),l_{3}\left(0.6\right)\right\}$
**PLNIS**	$\left\{l_{-3}\left(0.6\right),l_{-2}\left(0.2\right),l_{-1}\left(0.2\right)\right\}$	$\left\{l_{-3}\left(0\right),l_{-3}\left(0.6\right),l_{-1}\left(0.4\right)\right\}$

**Table 9:** $d\left(PLA_{i},PLPIS\right)$ and $d\left(PLA_{i},PLNIS\right)$ of each alternative.

Alternatives	$d\left(PLA_{i},PLPIS\right)$	$d\left(PLA_{i},PLNIS\right)$
A_1_	0.1589	0.1310
A_2_	0.2565	0.0198
A_3_	0.0610	0.2273
A_4_	0.2185	0.1006
A_5_	0.2342	0.1159

**Table 10:** Probabilistic linguistic relative closeness degree (PLRCD of each alternative from PLPIS.

Alternatives	A_1_	A_2_	A_3_	A_4_	A_5_
$PLRCD\left(PLA_{i},PLPIS\right)$	0.4518	0.0716	0.7884	0.3153	0.3310
